# Salivary pH as a marker of plasma adiponectin concentrations in Women

**DOI:** 10.1186/1758-5996-4-4

**Published:** 2012-02-03

**Authors:** Monique Tremblay, Yacine Loucif, Julie Methot, Diane Brisson, Daniel Gaudet

**Affiliations:** 1 Department of Medicine, Université de Montréal, ECOGENE-21 and Lipid Clinic, Chicoutimi Hospital, Saguenay, QC, Canada

**Keywords:** Salivary pH, Adiponectin, Cardiometabolic risk, Diabetes

## Abstract

**Background:**

Plasma adiponectin is a significant correlate of the pro-inflammatory cardiometabolic risk profile associated with obesity and type 2 diabetes. Salivary pH is influenced by several cardiometabolic risk components such as inflammation, oxidation and numerous oral and systemic health modulators, including the menopausal status. This study aimed to assess the association between plasma adiponectin concentrations and salivary pH in women according to the menopausal status.

**Method:**

Unstimulated saliva collection was performed in 151 Caucasian women of French-Canadian origin (53 premenopausal women (PMW) and 98 menopausal women (MW)). Student's t test, ANOVA and linear regression models were used to assess the association between plasma adiponectin concentrations and salivary pH.

**Results:**

Plasma adiponectin levels increased as a function of salivary pH in the whole sample and among MW (r = 0.29 and r = 0.36, p < 0.001). The proportion of the variance of plasma adiponectin levels explained by the salivary pH (R^2^) was 10.8% (p < 0.001). Plasma adiponectin levels progressively increased across salivary pH quartiles (p = 0.005).

**Conclusions:**

These results suggest that salivary pH is a significant correlate of plasma adiponectin levels in women. With the increasing prevalence of type 2 diabetes and obesity, new technologies should be developed to more easily monitor health status, disease onset and progression. Salivary pH, a simple, inexpensive and non-invasive measure, could be a very promising avenue.

## Introduction

According to the World Health Organization, by 2015 approximately 2.3 billion adults worldwide will be overweight and more than 700 million obese [1]. The burden of the associated cardiometabolic risk components, such as type 2 diabetes mellitus (T2DM), dyslipidemia and cardiovascular disease (CVD), is also set to grow rapidly. Not only is this epidemic progression observed in developed countries, but it increasingly affects developing countries too. Moreover, it has been estimated that by 2025, three out of four people with T2DM or other cardiometabolic risk components will be living in third world countries [2]. It is therefore mandatory to develop improved and more accessible screening tools in order to devise finer preventive and therapeutic strategies.

Adiponectin has been identified as a new biochemical marker of visceral fat accumulation and could represent a critical link between obesity and the cardiometabolic risk profile [3]. Adiponectin is an adipocyte-derived protein, highly abundant in plasma, with concentrations ranging from 5 to 30 μg/ml (0.01% of total plasma) [4]. Its levels correlate with the development of T2DM [5,6], dyslipidemia, hypertension [7] and CVD [8]. Adiponectin may also have different immune functions in several biological systems. It exerts anti-inflammatory, insulin-sensitizing and anti-atherosclerotic effects when secreted into the circulation [9]. Therefore, in the search for indexes of metabolic perturbations and proinflammatory status predisposing to a deteriorated cardiometabolic risk profile, the measurement of plasma adiponectin concentrations is increasingly studied. However, in the context of this epidemic progression of T2DM and obesity and in order to develop accessible tools without increasing the burden of healthcare costs, it is imperative to develop alternatives to the traditional measurement of blood markers. To meet this need, among the biofluids of the human body, the sampling of saliva is the most readily available and non-invasive method. It could thus be an interesting avenue.

Salivary function depends on its flow rate and composition. A healthy flow rate is critical for the maintenance of the whole body health. Saliva helps bolus formation by moistening food, protects the oral mucosa against mechanical damage, plays a role in preliminary digestion and has defense functions against pathogen microorganisms [10]. The saliva flow rate is also a modulator of salivary pH. At low flow rates, less bicarbonate is released, and pH decreases [11]. Salivary flow rates vary widely between subjects [12]. However, it remains quite constant during the different stages of life for a given individual. In fact, low secretors tend to stay in the low range, high secretors stay in the high range and so on [12]. On average, women tend to have lower flow rates than men. In addition, at the individual level, women seem to have more variation in their salivary pH as well. It has been suggested that hormonal fluctuations during events like puberty, menstruation, pregnancy and menopause could explain those differences [13]. Moreover, there is a noticeable decrease of unstimulated saliva after menopause [12]. The salivary flow rate is also affected by various cardiometabolic risk components [14]. Degenerative alterations in the acinar cells, which cause a decrease of the saliva flow rate and a diminution of salivary pH, are frequently observed among diabetic and dyslipidemic patients [15]. Hyposalivation has also been linked to obesity, aging and hypertension [12,16,17].

Interestingly, while the link between plasma glycaemia and salivary glucose concentrations seems weak, a significant correlation has been found between salivary and plasma adiponectin levels [18,19]. Adiponectin is produced by salivary gland epithelial cells where it might be implicated in the regulation of the local immune response [20]. Thus adiponectin could help to preserve a good salivary function and maintain the salivary pH. Besides, considering the influence of menopause on the salivary flow rate, this effect could be influenced by the menopausal status.

The aim of this study is therefore to assess the association between salivary pH and plasma adiponectin concentrations in women according to the menopausal status.

## Methods

### Subjects and clinical data

The study comprised a sample of 151 Caucasian women of French-Canadian origin followed at the Chicoutimi Hospital Lipid Clinic (Quebec, Canada). Two groups were formed according to the menopausal status. Postmenopausal status was defined by a self-reported menstrual status during clinical interviews or was automatically attributed to all women over 52 years or older at the time of the interview. The first group is composed of 53 premenopausal women (PMW) and the second group of 98 menopausal women (MW). Anthropometric variables were measured according to the procedures recommended by the Airlie Conference [21]. T2DM was diagnosed using the American Diabetes Association criterion, that is a 2-hour plasma glucose ≥ 200 mg/dl (11.1 mmol/l) during an oral glucose tolerance test (OGTT). The test was performed as prescribed by the World Health Organization, using a glucose load containing the equivalent of 75 g anhydrous glucose dissolved in water (Glucodex^® ^75 g) [22]. The project received the approval of the Chicoutimi Hospital Ethics Committee, in accordance with the Declaration of Helsinki.

### Biochemical analyses

Blood samples were obtained after a 12-hour overnight fast from the antecubital vein into vacutainer tubes containing EDTA. The HDL subfraction was obtained after precipitation of LDL (d > 1.006 g/ml) in the infranatant with heparin and MnCl_2 _[23]. Cholesterol, glucose and triglyceride (TG) levels were measured by enzymatic essays on a Multiparity Analyser CX7 (Beckman, Fullerton, CA, USA). Fasting plasma adiponectin concentrations were measured by ELISA (B-bridge International, Inc. San Jose, CA, USA). The intra- and inter-assay coefficients of variation were 4.00% and 18.98%, respectively. Plasma glycerol concentrations are an important intermediate of glucose and lipid metabolism. They were measured with an analyzer Technicon RA-500 (Bayer Corporation), and enzymatic reagents were obtained from Randox (Randox Laboratories).

### Saliva collection and pH measurement

Although stimulated saliva has generally been taken as the index of salivary function, the whole unstimulated saliva collection was chosen for the purpose of this study because it is the greatest contributor to the total salivary output [10,12]. All saliva samples were collected at least 2 hours after any food intake or smoking. Unstimulated saliva was allowed to accumulate in the floor of the mouth, and the subject then spat it out into a test tube during 10 minutes. The pH of the saliva sample was measured with Accumet Basic AB 15 pH Meter (Ottawa, Canada), and a 13-620-96 Micro pH electrode, 1.5" stem (127 mm) × 3 mm diameter with a pH range of 0 to 14 (Na+ < 0.1 N) and a selectable resolution to 0.1, 0.01 or 0.001 pH. The measurements were performed 3 times on each sample with a 0.01 resolution. The final result is the mean value of the measurements.

### Statistical analysis

Due to their skewed distribution, plasma TG, glycerol and adiponectin levels were log_10_-transformed before analyses. Geometrical means are presented. Differences in continuous variables were compared by either the Student's t test or ANOVA. Categorical variables were compared using the Pearson χ^2^. Linear regression models were constructed in order to investigate the relationship between salivary pH and plasma adiponectin concentrations, controlling for the effect of covariates significantly affecting both adiponectin concentrations and salivary pH. Effects of discrete variables were evaluated dichotomously. The reduced number of subjects in the study did not enable stratification for medication but corrections were applied in the different models. All statistical analyses were performed with the SPSS package (release 11.0, SPSS, Chicago III).

## Results

Subjects' characteristics are shown in Table 1. Differences in mean values between PMW and MW reached significance level (p < 0.05) for age, waist girth, salivary pH, glycerolemia, plasma TG, adiponectinemia and systolic blood pressure. Moreover, the percentage of T2DM among MW was almost twice the one of PMW (p = 0.018).

**Table 1:** Subjects' characteristics according to the menopausal status

	Pre-menopause (n = 53)	Menopause (n = 98)	p-value
Age (years)	47.9 ± 7.2	64.8 ± 7.6	**< 0.001**

BMI (kg/m^2^)	27.6 ± 5.6	29.3 ± 5.6	0.083

Waist girth (cm)	89.5 ± 12.6	94.9 ± 13.3	**0.018**

Salivary pH	6.63 ± 0.33	6.76 ± 0.32	**0.017**

Glycemia(mmol/L)	5.68 ± 2.11	6.04 ± 1.85	NS

Glycerol (mmol/L) *	0.07 ± 0.07	0.11 ± 0.05	**< 0.001**

TC (mmol/L)	5.78 ± 1.41	6.16 ± 1.14	0.072

HDL-C (mmol/L)	1.37 ± 0.49	1.40 ± 0.34	NS

LDL-C(mmol/L)^a^	3.76 ± 1.03	3.69 ± 0.86	NS

TG (mmol/L) *	1.35 ± 3.93	1.90 ± 3.15	**0.005**

Adiponectin (μg/m) *	6.22 ± 4.54	7.69 ± 4.66	**0.027**

Systolic BP	121.09 ± 17.37	133.61 ± 16.73	**< 0.001**

Diastolic BP	74.15 ± 8.84	72.77 ± 9.07	NS

Type 2 diabetes (%)**	22.6	41.8	**0.018**

Abbreviations: **BMI**: body mass index; **TC**: total cholesterol; **HDL-C**: high-density lipoprotein cholesterol; **LDL-C**: low-density lipoprotein cholesterol; **TG**: triglycerides; **BP: **Blood pressure. Mean ± SD. NS: p > 0.1 ^a ^n = 143

*P-values have been obtained after the log_10_-transpormation of the data. Geometric means are shown.

** χ-2 value

**Figure 1:**
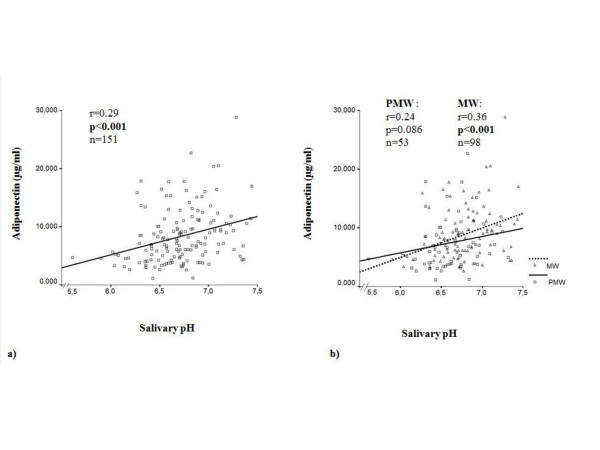
**a) Association between plasma adiponectin levels and salivary pH in the whole sample, adjusting for age; b) Association between plasma adiponectin levels and salivary pH in premenopausal women and menopausal women, adjusting for age**.

**Figure 2:**
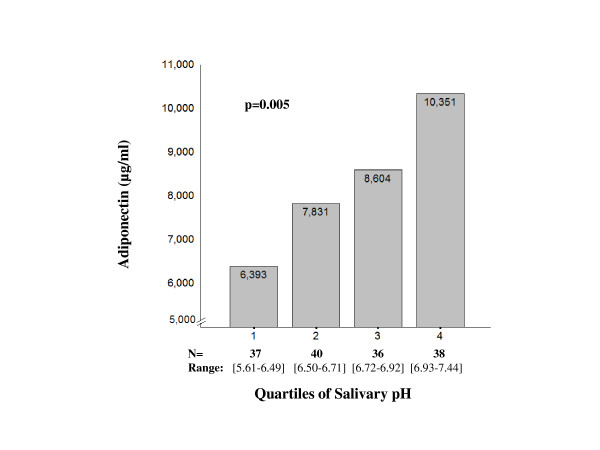
**Mean plasma adiponectin levels across quartiles of salivary pH in the whole sample, taking into account the effect of age**.

As shown in Figure 1, plasma adiponectin levels increased as a function of salivary pH in the whole sample and among MW (r = 0.29 and r = 0.36, p < 0.001). Although the correlation did not reach significance level in PMW, the same trend was observed (r = 0.24; p = 0.086). Plasma adiponectin levels progressively increased across salivary pH quartiles (p < 0.005) in the whole sample (Figure 2).

The proportion of the variance of plasma adiponectin levels explained by the variations of salivary pH (R^2^) was 10.8% (p < 0.001) in the whole sample (Table 2) and 13.0% (p < 0.001) in the MW (data not shown). The linear relation between salivary pH and plasma adiponectin levels remained significant even after including age, menopausal status, T2DM expression, waist circumference and body mass index (BMI) in the multivariate models (Table 2). When MW were analyzed separately, the linear relation followed the same trend. Moreover, these results remained significant even after the inclusion of hormone replacement therapy and lipid-lowering medication use to the models (data not shown).

**Table 2:** Linear regression analyses between salivary PH and plasma adiponectin levels in the whole sample

	Model 1	Model 2*	Model 3*	Model 4*	Model 5*	Model 6*
*R^2^*	**10.8**	**11.8**	**12.3**	**24.4**	**25.3**	**27.2**
*p*-value	**< 0.001**	**< 0.001**	**< 0.001**	**< 0.001**	**< 0.001**	**< 0.001**

**Salivary pH**						
*p-value*	**< 0.001**	**0.001**	**0.001**	**0.003**	**0.003**	**0.002**

**Menopause**						
*p*-value			0.404	0.405	0.337	0.208

**Type 2 diabetes**						
*p*-value				< 0.001	< 0.001	0.001

**Waist girth**						
*p*-value					0.202	0.021

**BMI**						
*p*-value						0.052

* Including age as covariate

Dependent variable: Log_10 _of adiponectin

## Discussion

In our study, we found that salivary pH is significantly associated with plasma adiponectin levels in women, particularly in MW. This association is independent of age, waist circumference and T2DM. Although numerous studies have shown correlations between serum and saliva levels for a wide range of molecular components, none has studied saliva from this simple perspective [14,24,25].

Alteration in blood adiponectin concentration has been linked to several metabolic disorders, and a low plasma level of adiponectin is a significant correlate of the pro-inflammatory cardiometabolic risk profile [26,27]. Adiponectin is a critical link between visceral adiposity, insulin resistance and CVD. Among all adipocytokines, it is the only one with a circulating concentration inversely proportional to adiposity [6]. As with the salivary flow, adiponectin production is inhibited by inflammation and oxidative stress, while medications used for several cardiometabolic risk components are known to increase its levels [28]. The same pattern is observed in our results. Low adiponectinemia is associated with a low salivary pH. The decrease of salivary pH could be due to a decrease in the salivary flow rate that may be associated with systemic inflammation. Alterations in saliva composition or flow rate may reflect secondary systemic changes related to diseases, medications or treatments. Those conditions may trigger an inflammatory status by dysregulation of the cytokine profile [29]. Interestingly, our results remain significant even after the inclusion of the diabetic status in the model. Diabetes is known to be associated with both adiponectin levels and salivary pH [16,27,30]. Diabetes is the most frequent metabolic disease associated with salivary hypofunction and thus with a decreased salivary pH [16]. Although the causal relationship remains unknown, many explanations have been proposed. It may be related to alterations in the major salivary glands similar to changes observed in the pancreas [14], or linked to autonomic neuropathies triggering a diminished response to normal salivary stimuli, or caused by the dehydration that often occurs in diabetic individuals [30]. The systemic inflammatory state has also been mentioned as a possible cause of hyposalivation in T2DM.

The plasma adiponectin level is influenced by a lot of variables and the numerous interactions between them. In this context, most of these variables account for only a small proportion of adiponectin variance so that their clinical impact may seem small when taken separately. However, when they are taken together, the proportion can rapidly rise. Although modest, the observed relationship between salivary pH and adiponectin could therefore be significant especially since the relationship between adiponectin and salivary pH remains significant even when other known covariables are included in the model.

Our study has some limitations. Although the cross-sectional nature of the study takes into account with great accuracy the metabolic conditions present before the evaluation of salivary pH and plasma adiponectin levels, previous conditions, like insulin resistance or hyperlipidemia, could have influenced their association. Indeed, the alterations of salivary glands, which may occur following dyslipidemia or diabetes, should be assessed in order to exclude any permanent deficiency or lesion of the acini. Another limitation is selection bias since participants were recruited among lipid clinic patients. This could have led to a higher prevalence of metabolic syndrome related components such as T2DM. Before any conclusion can be drawn about the use of salivary pH as a marker of metabolic disturbances, it is mandatory to reproduce these analyses in men and other populations with different phenotypes. Finally, the study does not give any information about the potential causal pathway that may be implicated. Since the salivary flow rate and pH remain relatively constant for a given individual during the different stages of life, salivary gland hypofunction is more commonly associated with concomitant diseases or daily use of drugs. Therefore, a study design with pre- and post-treatment analyses would add a lot of complementary data to the present study.

Despite these limitations, our study emphasized the important association between systemic and oral health. The results show that variations in salivary pH could provide a marker of metabolic dysregulation and may be associated with several elements of the cardiometabolic risk profile. The measurement of salivary pH does not need substantial professional and material resources, can be performed in any clinical setting and is inexpensive. It therefore opens the door to the development of an accessible screening tool for both developed and developing countries. Other components within saliva may provide additional clues on systemic health conditions. Besides, the development of new technologies may promote a wider use of salivary assays in the near future. The lower level of analytes is not a limitation anymore, because the salivary measurement of small molecules, such as peptides and cytokines, is now feasible. Moreover, there are compelling reasons to use saliva to monitor health and diseases. Saliva sampling is an inexpensive, non-invasive and easy-to-use method. The new, sensitive techniques may allow a better monitoring of the health status, disease onset and progression [25,31]. The evaluation of biomarker levels of T2DM and CVD could be performed directly from oral fluids, eliminating the need for blood sampling. Thereby the burden of healthcare costs could be reduced while increasing the accessibility to screening tools. However, much work needs to be done in identifying definitive, disease-associated salivary biomarkers. Many studies should validate the saliva-based test before it can be used for diagnosis applications.

## Conclusion

Saliva is still little used compared to plasma although it offers several opportunities in diagnostic and monitoring. With the emerging worldwide epidemic of T2DM, obesity and associated disorders, new technologies should be developed to more easily monitor health status, disease onset and progression. New accessible biomarkers that could be readily included in an integrated screening approach, with other non-invasive and accessible markers, should be identified. In this context, we think that salivary pH could be a very promising avenue.

## Competing interests

The authors declare that they have no competing interests.

## Authors' contributions and information

MT has conceived the study design, performed the data analysis/interpretation and written the manuscript. DB and DG have conceived the study design and revised the manuscript. YL and JM have revised the manuscript. All authors read and approved the final manuscript.
